# Racial Discrimination in Health Care Is Associated with Worse Glycemic Control among Black Men but Not Black Women with Type 2 Diabetes

**DOI:** 10.3389/fpubh.2017.00235

**Published:** 2017-09-12

**Authors:** Shervin Assari, Daniel B. Lee, Emily Joy Nicklett, Maryam Moghani Lankarani, John D. Piette, James E. Aikens

**Affiliations:** ^1^Department of Psychiatry, University of Michigan, Ann Arbor, MI, United States; ^2^Center for Research on Ethnicity, Culture and Health, School of Public Health, University of Michigan, Ann Arbor, MI, United States; ^3^Department of Health Behavior and Health Education, School of Public Health, University of Michigan, Ann Arbor, MI, United States; ^4^Prevention Research Center, School of Public Health, University of Michigan, Ann Arbor, MI, United States; ^5^School of Social Work, University of Michigan, Ann Arbor, MI, United States; ^6^Veterans Affairs Center for Clinical Management Research, Ann Arbor, MI, United States; ^7^Department of Internal Medicine, Michigan Medicine, Ann Arbor, MI, United States; ^8^Department of Family Medicine, Michigan Medicine, Ann Arbor, MI, United States

**Keywords:** Blacks, African-Americans, gender, racial discrimination, diabetes

## Abstract

**Background:**

A growing body of research suggests that racial discrimination may affect the health of Black men and Black women differently.

**Aims:**

This study examined Black patients with diabetes mellitus (DM) in order to test gender differences in (1) levels of perceived racial discrimination in health care and (2) how perceived discrimination relates to glycemic control.

**Methods:**

A total of 163 Black patients with type 2 DM (78 women and 85 men) provided data on demographics (age and gender), socioeconomic status, perceived racial discrimination in health care, self-rated health, and hemoglobin A1c (HbA1c). Data were analyzed using linear regression.

**Results:**

Black men reported more racial discrimination in health care than Black women. Although racial discrimination in health care was not significantly associated with HbA1c in the pooled sample (*b* = 0.20, 95% CI = −0.41 −0.80), gender-stratified analysis indicated an association between perceived discrimination and higher HbA1c levels for Black men (*b* = 0.86, 95% confidence intervals (CI) = 0.01–1.73) but not Black women (*b* = −0.31, 95% CI = −1.17 to −0.54).

**Conclusion:**

Perceived racial discrimination in diabetes care may be more salient for glycemic control of Black men than Black women. Scholars and clinicians should take gender into account when considering the impacts of race-related discrimination experiences on health outcomes. Policies should reduce racial discrimination in the health care.

## Introduction

Discrimination, defined as differential treatment of members of a group by both individuals and social institutions (1), has adverse effects on physical health (2–5) and mental health (6–12). A particular type of discrimination, racial discrimination, entails targeting racial and ethnic minorities such as Blacks (4, 11, 13–19). In addition, Black men in general (20, 21) report the higher levels of racial discrimination than Black females.

Scientific evidence linking racial discrimination to poor health is overwhelming (13, 22, 23–27). Scholars have attributed this relationship to different mechanisms, including psychological and physiological stress responses (28), unhealthy coping behaviors (4, 29, 30), and lower health care utilization (31, 32). Most of this research has focused on the interpersonal experience of generalized racial discrimination, with less emphasis on perceived discrimination specific to the health care setting (33). At the same time, racial minorities who deny that racism exists are actually at an increased risk for adverse health outcomes (34–36) and lower health care utilization (37).

As patients with diabetes mellitus (DM) have frequent encounters with healthcare facilities, discrimination in the health care setting may be particularly important for racial minority groups with DM (38–40). That is, perceived health care discrimination is associated with worse health outcomes, poorer interpersonal communication with physician, higher hemoglobin A1c (HbA1c) levels, greater number of diabetes symptoms, and poorer physical functioning (39). This effect may be in part due to health behaviors and access to care (41).

Research on discrimination and diabetes is limited (38–40). In one study, DM patients reporting higher levels of healthcare discrimination were less likely to receive HbA1c tests and eye exams (42). In another study, patients with DM who self-reported healthcare discrimination also had lower quality diabetes care (40). Finally, among patients with DM, perceived discrimination was associated with lower quality patient/provider communication and worse glycemic control (39).

Some evidence, mostly derived from community surveys, suggests that gender may modify the effect of perceived discrimination upon health, with a stronger effect for men than women (23, 24, 43–46). Although the mechanisms underlying gender differences in the discrimination and health link is still unknown, masculinity, social hierarchy, and expectation for dominance may have a role (47). Further, even less is known about gender differences in the health effects of perceived racial discrimination in the health care settings. In particular, gender differences in actual and perceived racial discrimination may help explain why discrimination may be more damaging for men than women (47).

The current study had two aims: (1) to investigate gender differences in perceived racial discrimination in health care among Black patients with DM and (2) to compare Black men and women for the relationship between perceived discrimination and patients’ glycemic control. In line with prior documentation that Black men report greater interpersonal discrimination than Black women (48, 47), we expected Black men to experience more perceived discrimination in health care than Black women do. In addition, as considerable evidence suggested that the relation between discrimination and health may be different between men and women (23, 24, 43–45), we expect that perceived health care discrimination would be more strongly associated with glycemic control for Black males than for Black females.

## Materials and Methods

### Design and Setting

This cross-sectional study was conducted in an outpatient setting in a large Midwestern urban health care system. Data came from the Racial Differences in Diabetes-Depression Comorbidity (RDDDC) study, 2005–2007.

### Ethics

The research protocol followed the tenets of the Declaration of Helsinki. The study protocol was approved by the Institutional Review Board. All participants provided comprehensive written informed consent. Data were kept confidential.

### Participants and Sampling

The study used a consecutive sampling strategy. The parent study identified eligible patients from the administrative and clinical databases (23, 49). In order to participate, patients needed to (1) be between 18 and 80 years of age, (2) be ability to complete self-report instruments, (3) have a recorded diagnosis of type 2 DM, and (4) self-identify as either non-Hispanic Caucasian/White or African-American/Black. The current study was limited to the subsample of 163 who identified as Black or African-American. Diagnosis of DM was based on at least one of the following criteria: (1) positive history of hospitalization with a DM-related ICD-9 code (250.*x*, 357.2, 362.0, or 366.41), (2) two or more outpatient visits with a DM-related ICD-9 code, or (3) prescription for a glucose control medication or monitoring supplies. Use of ICD-9 was the standard practice when the data were collected. The study excluded individuals with type 1 DM through chart review and telephone screening conducted by research staff.

### Enrollment of Patients and Response Rate

Eligible patients were mailed a study invitation letter followed by a recruitment telephone call from research staff for further screening and enrollment. Data for the present analysis were collected at a single time point.

#### Sociodemographic Factors

The study collected data on self-reported age and gender (male vs. female). Socioeconomic status (SES) was assessed using the U.S. Census Bureau Index of SES, adjusted for the regional Consumer Price Index at the time of data collection. Occupation, educational attainment, and income are all related, but none of them by adequately indicates SES. The SES score is based on occupation and educational attainment of the chief income recipient in the family and current family income (50, 51).

#### Self-rated Health (SRH)

Participants rated their own health on a five-point Likert-type scale ranging from “excellent” to “poor.” This single item measure has been widely used in health research, including studies on race, gender, DM, and SRH (43, 44, 52–55). A higher SRH score indicates poorer SRH.

#### Perceived Racial Discrimination in Health Care

An 8-item modified version of the Chen et al. measure (33) was used to assess patients’ general belief about racism in the health care system, including unfair treatment, poor access to services, low quality of care, and unequal costs. Items are rated using a combination of 3- and 4-point Likert scales, and we calculated the mean score for data analysis. Thus, scores potentially ranged from 1 to 4, with higher scores reflecting greater perceived racial discrimination in health care settings. This measure has been frequently used to measure perceived discrimination in health care and, in our sample, demonstrates good reliability for Black men and women (α_Overall_ = 0.846, α_Males_ = 0.821, α_Females_ = 0.861).

Table A1 in Appendix shows original items and our modified version. The modification was limited to omitting the only positively worded item “Do you think most African-Americans (Latinos) receive the same quality of health care as whites?” which significantly reduced the reliability of the measure, as all other items measure presence of racism and this item measures lack of racism in the health care.

#### Glycemic Control

On the same day that the self-report measures were completed, glycemic control (HbA1c) was evaluated with the DCA 2000 (GMI, Ramsey, MN, USA), which analyzes capillary blood samples through a monoclonal antibody method.

### Statistical Analysis

Data were analyzed using Stata 13.0 (Stata Corp., College Station, TX, USA) and Statistical Package for Social Sciences v.20 (SPSS 20). First, we tested normality of distribution of our numerical variables. Descriptive statistics such as means, SDs, and frequencies were used to describe the primary variables. Pearson correlations were examined to assess zero-order correlations. Multivariable linear regression models were used to predict the outcome of HbA1c as a function of perceived racial discrimination. The initial full model included as predictors age, SES, diabetes duration, and insulin. Coefficients were not significant for diabetes duration and insulin (*p* > 0.05) and were dropped from the final model. The final model included age, gender, and perceived discrimination as predictors of HbA1c. Model 1 only included main effects, and Model 2 included an interaction between gender and perceived discrimination. In secondary analyses, we examined the relationship between discrimination and HbA1c within subsamples of Black men and Black women. Unstandardized beta coefficients, 95% confidence intervals (CI), and *p*-values were reported. A *p*-value of 0.05 or less was interpreted as statistically significant.

## Results

Table 1 provides descriptive statistics by gender and in the pooled sample. Black men reported significantly higher levels of perceived racial discrimination in health care than Black women did (2.73 ± 0.60 vs. 1.39 ± 0.62, *p* < 0.05, independent samples *t*-test). Black men also had higher HbA1c levels on average compared to Black women; however, the difference was not significant (*p* > 0.05, independent samples *t*-test). Although Black women had slightly higher education levels than Black men (*p* > 0.05, independent samples *t*-test), household income was significantly higher for Black men (82.84 ± 23.80 vs. 67.14 ± 29.86, *p* < 0.05, independent samples *t*-test) (Table 1).

**Table 1:** Descriptive statistics for pooled and gender-stratified samples.

	All	Men	Women
	
	Mean (SD)	Mean (SD)	Mean (SD)
Age (years)	54.47 (8.18)	53.29 (9.02)	55.80 (6.95)
Income (USD 1000)*	75.29 (27.93)	82.84 (23.80)	67.14 (29.86)
Education	62.00 (24.86)	61.11 (24.12)	63.08 (25.89)
Diabetes duration (years)	10.25 (6.63)	10.63 (6.74)	9.83 (6.51)
SRH	3.19 (0.84)	3.04 (0.87)	3.36 (0.79)
Racial discrimination in health care*	1.39 (0.62)	2.73 (0.60)	2.50 (0.62)
HbA1c	7.87 (1.94)	7.94 (1.91)	7.79 (1.98)

*SES, socioeconomic status; HbA1c, hemoglobin A1c; SRH, self-rated health; USD, United States Dollars*.

*Higher score for SRH is indicative of worse health*.

*Education variable is centered*.

*p < 0.05 using independent samples t test.

Table 2 displays zero-order correlations for the pooled and gender-stratified samples. Notably, racial discrimination in health care was associated with HbA1c among Black men but not Black women. Lower age was significantly associated with receiving insulin. Age, education, income, and DM duration were not correlated with HbA1c.

**Table 2:** Pearson correlations for pooled and gender-stratified samples.

	1	2	3	4	5	6	7	8
**Pooled sample**								
1. Age	1.00	−0.23**	0.07	0.16*	−0.21**	0.06	−0.02	−0.30**
2. Income		1.00	0.22*	−0.09	0.16^#^	−0.16*	0.05	0.08
3. Education			1.00	−0.18*	0.02	−0.23**	0.08	−0.09
4. Diabetes duration				1.00	0.21**	0.10	−0.11	0.07
5. Insulin					1.00	0.10	0.12	0.11
6. SRH						1.00	0.13	0.10
7. Discrimination in health care							1.00	0.04
8. HbA1c								1.00
**Black men**								
1. Age	1.00	−0.32**	0.15	0.13	−0.27*	−0.01	−0.20	−0.30*
2. Income		1.00	0.16	−0.02	0.29*	−0.19^#^	0.02	0.10
3. Education			1.00	−0.24*	0.08	−0.38**	0.03	−0.09
4. Diabetes duration				1.00	0.29*	0.18	−0.04	0.12
5. Insulin					1.00	−0.01	0.21^#^	−0.08
6. SRH						1.00	0.19	0.11
7. Discrimination in health care							1.00	0.29*
8. HbA1c								1.00
**Black women**								
1. Age	1.00	−0.09	−0.06	0.25*	−0.19^#^	0.09	0.16	−0.32**
2. Income		1.00	0.32*	−0.21^#^	0.11	−0.06	0.19	0.03
3. Education			1.00	−0.10	−0.06	−0.08	0.12	−0.09
4. Diabetes duration				1.00	0.13	0.04	−0.14	0.01
5. Insulin					1.00	0.19	0.02	0.29*
6. SRH						1.00	−0.03	0.10
7. Discrimination in health care							1.00	−0.18
8. HbA1c								1.00

*SES, socioeconomic status; HbA1c, hemoglobin A1c; SRH, self-rated health*.

*Higher score for SRH is indicative of worse health*.

*Education is centered*.

*^#^p < 0.1*.

*p < 0.05.

**p < 0.05.

As shown in Table 3, perceived racial discrimination in health care did not have a main effect on HbA1c in the pooled sample (*b* = 0.20, 95% CI = −0.41 to 0.80). We, however, found a significant interaction between gender and racial discrimination in health care on HbA1c (*b* = −1.20, 95% CI = −2.40 to 0.00), suggesting a stronger association between racial discrimination in health care and HbA1c for Black men than Black women. Age was significantly and negatively associated with HbA1c in the pooled sample of Blacks (*b* = −0.08, 95% CI = −0.12 to −0.03).

**Table 3:** Summary of regression analyses in the pooled sample.

	*b*	95% CI	*b*	95% CI
		
	Model 1		Model 2	
	Main effects in the pooled sample		Main effects and interactions in the pooled sample	
Gender (women)	−0.01	(−0.76 to 0.73)	−3.19^#^	(−6.47 to 0.09)
Age (years)	−0.08**	(−0.12 to −0.03)	−0.07**	(−0.12 to −0.02)
Discrimination	0.20	(−0.41 to 0.80)	0.83^#^	(0.04 to 1.70)
Gender × discrimination	–	–	−1.20*	(−2.40 to −0.01)

*Outcome: HbA1c, hemoglobin A1c*.

*Discrimination: Perceived discrimination in health care*.

*b: Unstandardized regression coefficient*.

*^#^p < 0.1*.

*p < 0.05.

**p < 0.05.

Table 4 shows the results of the gender-stratified regression models. Perceived discrimination in health care was positively associated with higher HbA1c for Black men (*b* = 0.86, 95% CI = 0.00 to 1.73) but not for Black women (*b* = −0.31, 95% CI = −1.17 to 0.54). Age was significantly and negatively associated with HbA1c in Black women (*b* = −0.10, 95% CI = −0.18 to −0.01). Although in the same direction, the association between age and HbA1c was marginal in Black men (*b* = −0.05, 95% CI = −0.11 to 0.01).

**Table 4:** Summary of gender-stratified regression models.

	*b*	95% CI	*b*	95% CI
		
	Model 3		Model 4	
	Men		Women	
Age (years)	−0.05	(−0.11 to 0.01)^#^	−0.10	(−0.18 to −0.01)*
Discrimination	0.86	(0.01 to 1.73)*	−0.31	(−1.17 to 0.54)

*Outcome: HbA1c, hemoglobin A1c*.

*Discrimination: perceived discrimination in health care*.

*b: unstandardized regression coefficient*.

*^#^p < 0.1*.

*p < 0.05.

## Discussion

We found that among Black DM patients, men reported more racial discrimination in health care settings than women did. Our findings also suggest that perceived health care discrimination is associated with poorer glycemic control among Black men, but not among Black women. These findings collectively indicate the importance of incorporating the interplay between race and gender when considering the health consequences of discrimination.

Our first finding is consistent with prior research that delineates the gendered nature of racial discrimination experience in the lives of Blacks (20, 21). Similar findings have been reported in other contexts such as general population (56–61). Among Blacks, male children and adolescents receive more racial socialization messages that prepare them for these race-related encounters (62–64). Gendered racial socialization, in turn, may result in gender differences in race-related attitudes, identity, and experiences later in life (65, 66). As a result, Black men may be more aware of racism and discrimination across multiple settings (e.g., school, work, health care).

Our second finding, the stronger association between perceived discrimination and glycemic control for Black men than Black women, is also supported by the literature (24, 67, 68). In a study on young Black adults, racial discrimination was associated with depression and anxiety symptoms among males but not females (67). Similarly, a prior study of Arab American adults showed that discrimination was strongly associated with psychological distress among males, but not among females (24).

These findings suggest that Black men and Black women may differently cope with environmental stressors, including discrimination and neighborhood stress. While environmental stressors such as discrimination better predict depression for Black men than for Black women (68–70), neighborhood stress and fear of violence have shown to have stronger effects on obesity for Black women than men (68, 71, 72). This gender-specific health consequences of environmental stressors may be due to Black men’s and women’s tendency to use substances (e.g., alcohol use) and over-eat in response to environmental stress (28, 73). Gender differences in coping (74–76) are also well documented.

There have been two primary explanations offered for the finding that Black men report higher rates of discrimination than Black women (20, 21). The first explanation, called the subordinate male target hypothesis, argues that Black men are subject to more experiences of discrimination (47). The second explanation attributes males’ higher perceived discrimination to measurement bias (47, 77). Ifatunji and Harnois (47) found that subordinate male hypothesis (i.e., that subordination is causing more distress for men than women) explains the gender gap in everyday discrimination.

We tested gender as an effect modifier for the hypothesized effects on glycemic control of perceived discrimination. Prior work suggests that discrimination is more strongly associated with health outcomes for men than for women (24, 45). Moreover, even among men, hejamonic and traditional masculine ideologies may alter how discrimination shapes health (78–80). This suggests that masculine beliefs may explain why Black men report more discrimination in health care setting than women. In addition to gender and masculinity, education (22), social class (81), racial attribution (82), and racial identity (4, 83–85) also probably influence the association between discrimination and health outcomes.

Our findings, which reveal gender differences in the deleterious effect of racial discrimination in health care settings for Blacks with DM, have valuable research and clinical implications. Clinically, racial discrimination in the health care system should be considered as a structural risk factor with health implications for Blacks, particularly Black men. Preventive interventions to address racial discrimination in health care settings should be considered a strategic goal toward elimination of health disparities. Cultural competency training for health care providers is just one of many ways to reduce social inequities in health outcomes (86–91).

Despite the fact that racial disparities in health outcomes and self-care behaviors among adults with type 2 DM is well known (80), little is known regarding the mechanisms behind such racial differences in DM outcomes. The present findings extend this limited understanding by characterizing gender differences within Blacks. Based on our findings, racial discrimination against Blacks particularly Black men may play an important role for DM outcomes.

Current finding that male Black patients with type 2 DM perceive more racial discrimination in health care settings than Black women with the same condition, and perceived racial discrimination in health care settings has an impact in Black men’s overall glycemic control has policy and clinical implications for reducing racial disparities in DM outcomes. The link between the perception of racial discrimination and the poor glycemic control is in line with previous reports on associations between depression and psychological distress and glycemic control (49, 92). Findings are relevant as racial disparities exist in DM outcomes particularly for Blacks (93, 94).

Additional research is needed to understand how racial discrimination in health care settings influences the health care utilization and self-care of Black men. Future research should explore the role of class, masculinity, maladaptive coping, health behaviors, health care use, medication adherence, mental health, racial attribution, and racial identity to explain why Black men are more vulnerable to health consequences of discrimination. Future research should also investigate how exposure to the full spectrum of discrimination influences health of Blacks. Researchers should also direct efforts and resources toward understanding system level determinants of discrimination as experienced by Black patients in health care systems. This study, alongside others (95, 96), suggests that Black men are particularly sensitive to discrimination encounters in health care settings. By cultivating a body of research that reflects racial discrimination in health care settings, scholars and policy makers will be able to design effective policies and interventions to reduce individual- and structural-level discrimination within and beyond the health care setting.

This study is subject to a number of limitations. First, due to a cross-sectional design, we cannot interpret the observed associations as causal in nature. Second, mental health and depression were not measured, which could be confounders or underlying mechanisms for the link between perceived discrimination and glycemic control. Future research should replicate this study using a wider breadth of explanatory factors such as coping, racial identity, health care use, and satisfaction with care. Third, we did not measure frequency of exposure to discrimination and only perceived discrimination and racism in health care. Fourth, although Blacks face similar challenges at least in several other countries in the world, the history of slavery in the United States makes U.S. different from other countries regarding Blacks historical experience of racism and discrimination. U.S. health care system is also different from many other countries. As a result, it is not clear whether the results are generalizable to Blacks living in other countries. Despite these limitations, the study used an innovative conceptual model of discrimination and health that contributes to the growing body of research on racial health disparities.

In summary, we found that among Black patients with DM, men perceived more racial discrimination in health care settings than women and suffered in their glycemic control as a result. The findings from this study highlight the potential deleterious effects of discrimination health care settings as a pathway linking of racial disparities to health outcomes. To identify these methods, however, future research should focus on identifying potential mechanisms (e.g., patient–provider interactions, language, and class) that may further explain the link between racial discrimination and health. Anti-racist societal actions and anti-discrimination policies are needed in order to achieve more social equality.

## Ethics Statement

The University of Michigan Institutional Review Board approved the study protocol for all years of data collection and all participants provided consent for participation in the study.

## Author Contributions

This analysis was designed and performed by SA, who also contributed to drafts of the manuscript. JP and JA designed the main study, acquired the data, and contributed to all drafts of this manuscript. DL and EN also contributed to the drafts and conducted the revision. All authors confirmed the final version of the manuscript.

## Conflict of Interest Statement

The authors declare that the research was conducted in the absence of any commercial or financial relationships that could be construed as a potential conflict of interest.

## Appendix

**Table A1:** Chen measure of perceived racial discrimination in health care.

How often do you think our health care system treats people unfairly based on what their race or ethnic background is? How often do you think a person’s race or ethnic background affects whether they can get routine medical care when they need it? Is racism a major problem in health care? How often do you think racism occurs when a patient and doctor are of different racial or ethnic backgrounds? How often does racism occur if the patient and doctor are of the same racial or ethnic background? Do you think most African-Americans (Latinos) receive the same quality of health care as whites?^a^ For the average African-American (Latino), how big a problem is being able to afford the cost of health insurance and medical care? For the average African-American (Latino), how big a problem is having enough physicians and other health providers near where they live? For the average African-American (Latino), how big a problem is having difficulty getting care because of their race or ethnic background?

*^a^Item omitted in this study because of the positive wording (the item measured lack of racial discrimination)*.
